# Supplementary material for: Acceptance and compliance with micronutrient powder (MNP) among children aged 6–23 months in northern Nigeria
Source: PLOS Glob Public Health. 2022 Oct 17;2(10):e0000961. doi: 10.1371/journal.pgph.0000961 (PMC10022258; doi:10.1371/journal.pgph.0000961)
Supplement: S1 Dataset — (ZIP) [file pgph.0000961.s008.zip › S8_Dataset/Spot Checks.zip!/Spot Checks/07072015_A_GA_AS_2_SC.pdf]

SPOT CHECK RECORDING FORM

State: Adamawa LGA: ____Ganye___ Community: _Ganye 1_

Name of caregiver (female): Rejoice Kefas Yakubu

Name or caregiver (male): ………………………

Sex of index child: _Male___ Age of index child (in months): _ 7 months_______

Spot Check Information

1. **Date of MNP distribution to household** (circle option below based on LGA name above)**:**
   - **Kebbi:** BirninKebbi&Jega LGA: **June 16**
   - **Kebbi:** Danko-wasagu: **June 17**
   - **Adamawa:**Song, Ganye: **(June 18)**
   - **Adamawa:**Guyuk **June 19**
2. **Date of Spot Check Observation: ____7/7/2015_____**
3. **Number of sachets remaining**(Ask the caregiver to show you and count the specific number of sachets remaining)**:**

   42

4. **Number of sachets that should remainusing one MNP sachet per day** (Analyst only)**:**

   42

5. **Number of sachets remaining / number of sachets should be remaining**(Analyst only)**:**

   _____42___ / ___42______ = __100__ %

Probes

Question 1. You have **XX** MNP sachets remaining. Explain how easy or difficult it was to use the MNP product on a daily basis for your child 6 – 23 months? (Circle one answer)

Very Difficult **Difficult** **Easy** **Very Easy**

Question 2. Describe any specific challenges that you faced that made the MNP difficult to use on a daily basis?
I do not face any challenge in using this product on a daily basis. Everything is going normal. The only challenge is that the product does not dissolve quickly and when poured into kunu it creates lumps until I mix and mix for 3 to 4 minutes.

Question 3. Describe specific aspects of the MNP product that you or your child enjoyed that made it easier to use on a daily basis? The aspect that I enjoy most is the fact that my child does not reject the product and the cerelac I use in giving him. That makes my job as a mother a lot easier because my child comply by taking the product daily.

Question 4. What questions do you have about the MNP based on your experience using it?

Why is it that the product does not have the instructions about side effects and who shouldn't take the product. What can the product cure or what illness this product for. What if a mother skips two or three days can she continues?

Question 5. What specific recommendations do you have that we can do as a program to make the MNP easier to use on a daily basis?

The product should come with a pamphlet to indicate the way the product should be used and what the product stands for. It should be written clearly for those that can read to know how and when to use the product and know with what food to use with it. When it is clearly written caregivers that cannot read would give it to someone that can read and interpret it to them because when they are told only at the hospital or during home visits they may forget it.
